# Supplementary material for: Chemiluminescent screening of specific hybridoma cells via a proximity-rolling circle activated enzymatic switch
Source: Commun Biol. 2022 Apr 4;5:308. doi: 10.1038/s42003-022-03283-2 (PMC8979942; doi:10.1038/s42003-022-03283-2)
Supplement: Supplementary file 2 — Supplementary Information [file 42003_2022_3283_MOESM2_ESM.pdf]

Supporting Information

Chemiluminescent screening of specific hybridoma cells via a proximity-rolling circle activated enzymatic switch

Hang Ao, Weiwei Chen, Jie Wu✉, Wencheng Xiao & Huangxian Ju✉

State Key Laboratory of Analytical Chemistry for Life Science, School of Chemistry and Chemical Engineering, Nanjing University, Nanjing 210023, China.

✉email: wujie@nju.edu.cn; hxju@nju.edu.cn.

1. Supplementary Tables

Supplementary Table 1. Sequences of oligonucleotides used in this work (from 5' to 3'). The regions of the complementary sequences between oligonucleotides were shown in different colors and underline.

**DNA**	**Sequences**
DNA 1	TGAGGTAGTATGTCAGGACTTTTTTTATCACATCAGGCTCT ATGCTATTG-SH
DNA 2	HS-TACGTCCAGAACTTTACCCATCTTTTTTGTCCTGTTGTG TATAGTTGGATGCAA
Primer	TGAGGTAGTATGTTGTATAGTT
Padlock	Phosphate-ATACTACCTCACTCAATTCTGCTACTGTACTACAA CTATACAAC
Block	TTGCATCC AACTATACAACATACTACCTCA

Supplementary Table 2. Comparison of proposed assay with previously reported methods using different amplification strategies for antibody detection.

Assays*	Strategies	Detection	LOD	Reference
FL	Steric strain induced allosteric of hairpin structure	Homogenous	nM level	1, 2
FL	Steric hindrance inhibition of strand displacement	Homogenous	5.6 nM	3
FL	Proximity binding-induced strand displacement	Homogenous	nM level	4, 5
FL	Proximity induced hybridization chain assembly	Homogenous	3.2 nM	6
EC	Steric hindrance inhibition of strand binding	Homogenous	10 nM	7
EC	Proximity binding-activated DNA walker	Inhomogeneous	0.3 nM	8
EC	Proximity induced strand hybridization	Inhomogeneous	1 nM	9
ECL	Steric strain triggered cycling strand displacement	Inhomogeneous	6.7 pM	10
Colorimetry	Steric strain induced enzymatic recovery	Homogenous	100 pM	11
CLA	Proximity-rolling circle activated enzymatic switch	Homogenous	1 pg mL^{-1} (18 fM)	This work

*FL: fluorescent detection, EC: electrochemical detection, ECL: electrochemiluminescence detection, and CLA: chemiluminescent assay.

Supplementary Table 3. Recovery results of PCSK9-Ab in supernatant of hybridoma cells.

**Samples**	**Found (pg mL^{-1})**	**Added (pg mL^{-1})**	**Found after the addition (pg mL^{-1})**	**Recovery (%)**	**RSD (%)***
1	2.07	20	21.4	96.7	6.85
2	4.42	20	20.5	80.4	2.53
3	24.0	20	48.9	124.5	14.9
4	< LOD	20	19.9	99.5	3.19
5	< LOD	20	21.9	109.5	6.51

*RSD is calculated based on three parallel measurements.

2. Supplementary Figures

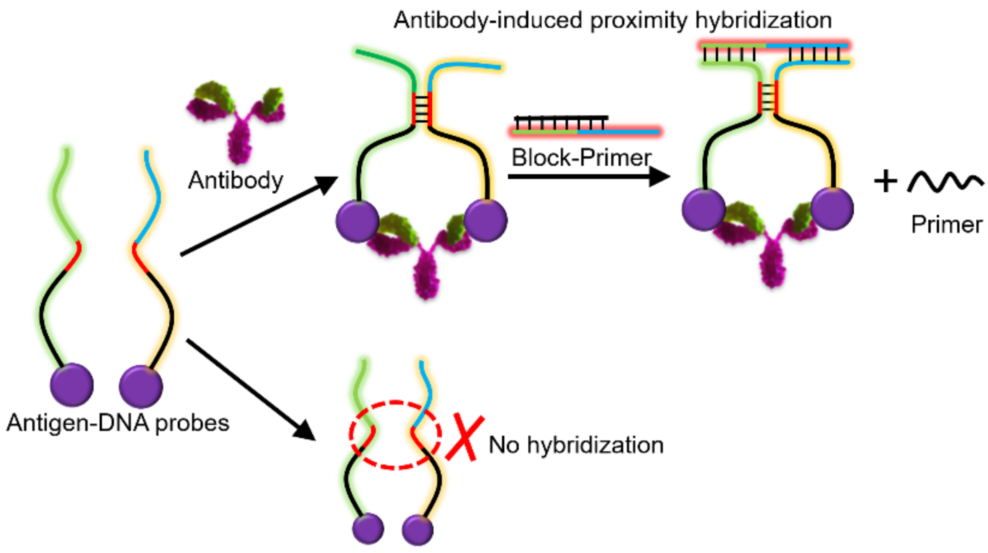

Supplementary Figure 1. Illustration of the antibody-induced proximity hybridization.

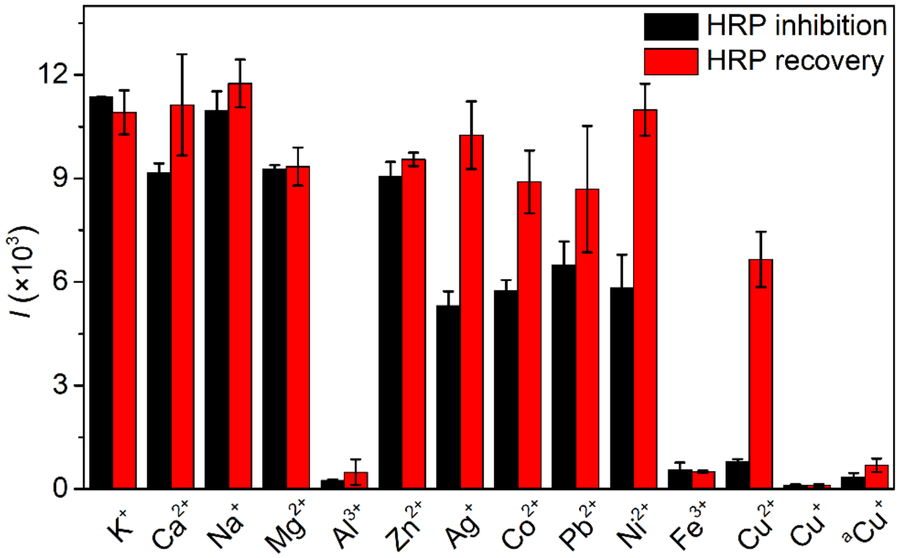

Supplementary Figure 2. HRP activity inhibition with metal ions (black columns) for 30 min and then recovery with PPi for 5 min (red columns). The concentrations of HRP, metal ions, PPi, luminol and H_2O_2 were 0.2 μg mL^{-1}, 120 μM, 2 mM, 0.2 mM and 1 mM, respectively. $^{a}Cu^{+}$ represented the concentration of Cu^{+} was 4 μM. Error bars were estimated from three parallel experiments.

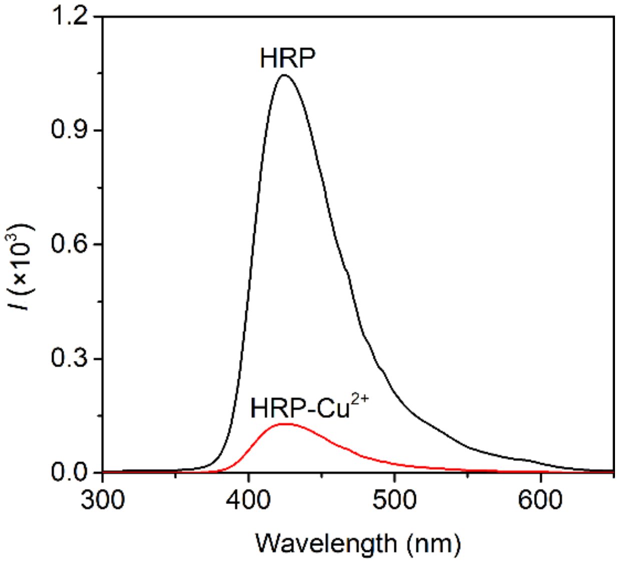

Supplementary Figure 3. Chemiluminescence spectra of 0.2 μg mL^{-1} HRP and HRP-Cu^{2+} in presence of 0.2 mM luminol and 1 mM H_2O_2.

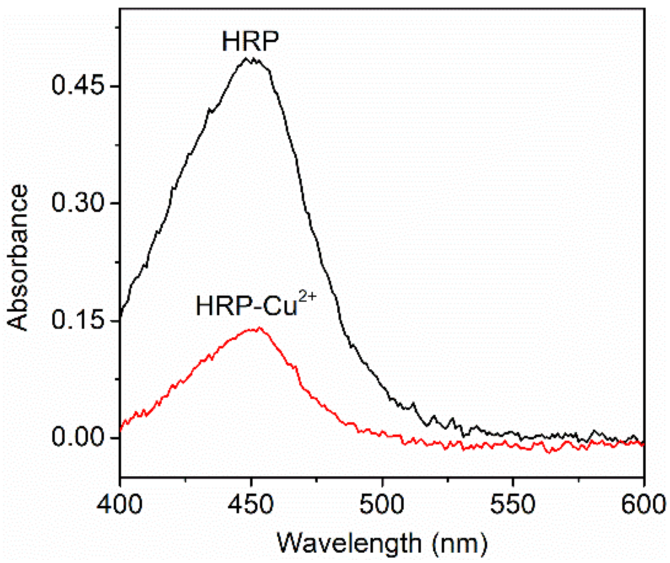

Supplementary Figure 4. Absorption spectra of 50 ng mL^{-1} HRP and HRP-Cu^{2+} added in TMB Kit for 30 min.

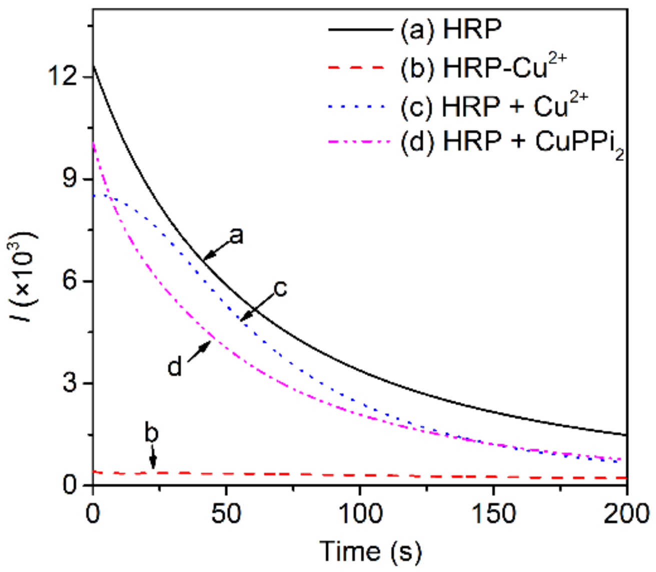

Supplementary Figure 5. Kinetic curves of (a) 0.2 μg mL^{-1} HRP, (b) 0.2 μg mL^{-1} HRP-Cu^{2+}, (c) the mixture of 0.2 μg mL^{-1} HRP and 0.12 mM Cu^{2+} and (d) the mixture of 0.2 μg mL^{-1} HRP and 0.12 mM $CuPPi_2$ in presence of 0.2 mM luminol and 1 mM H_2O_2.

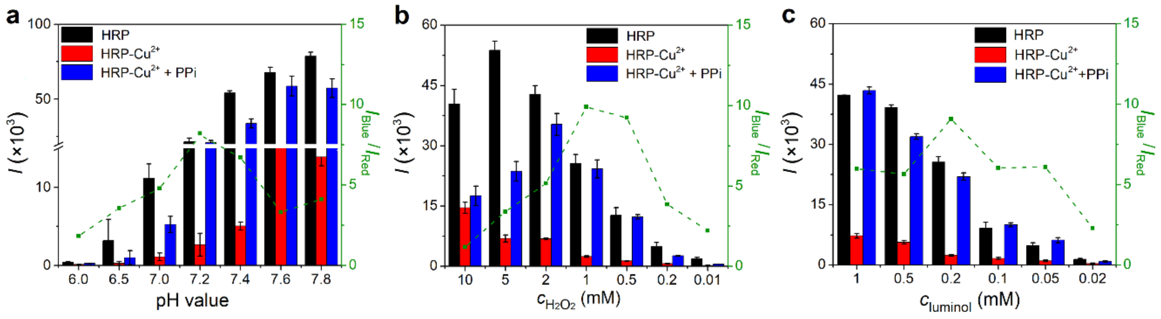

Supplementary Figure 6. Effects of **a** pH, **b** H_2O_2 concentration and **c** luminol concentration on chemiluminescent intensity of 10 mM pH 7.2 (**b**,**c**) Tris-HCl containing 0.2 mM luminol (**a**,**b**), 1 mM H_2O_2 (**a**,**c**) and 0.2 μg mL^{-1} HRP or 0.2 μg mL^{-1} HRP-Cu^{2+} in absence or presence of 2 mM PPi. Error bars were estimated from three parallel experiments.

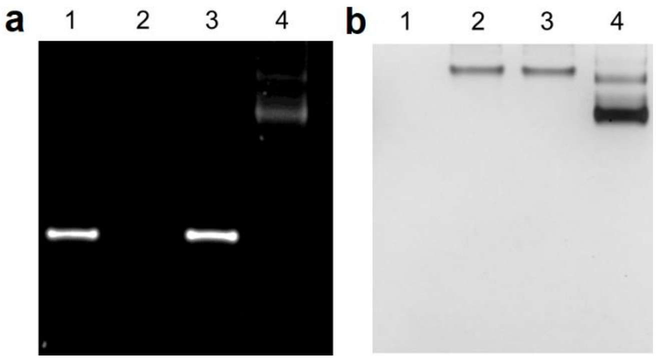

Supplementary Figure 7. a Native PAGE and **b** protein-staining images of DNA 1 (line 1), PCSK9 (line 2), mixture of DNA 1 and PCSK9 (line 3), and PCSK9-DNA 1 probe (line 4).

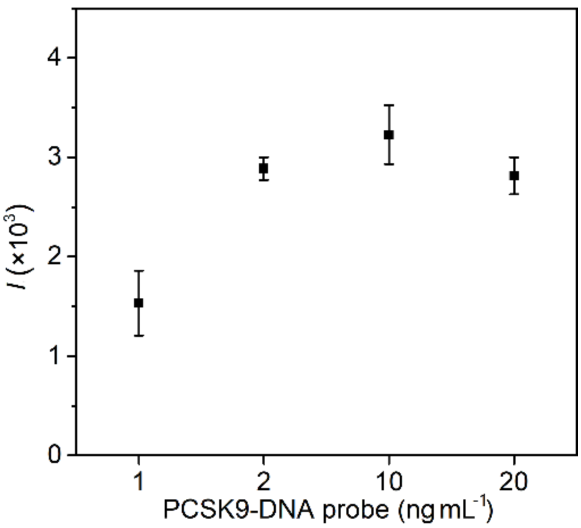

Supplementary Figure 8. Chemiluminescent signals of 10 mM pH 7.2 Tris-HCl containing 0.2 μg mL^{-1} HRP-Cu^{2+}, 0.2 mM luminol and 1 mM H_2O_2 after adding the reaction mixtures for 5 min. The reaction mixtures contain 40 nM block-primer, 0.12 μM padlock, 0.25 U μL^{-1} splint R, 0.01 U μL^{-1} phi 29, 0.4 mM BSA, 0.8 mM dNTP, 0.2 ng mL^{-1} PCSK9-Ab and PCSK9-DNA probes at 1, 2, 10 or 20 ng mL^{-1} for 1-h incubation at 37 ^{o}C. Error bars were estimated from three parallel experiments.

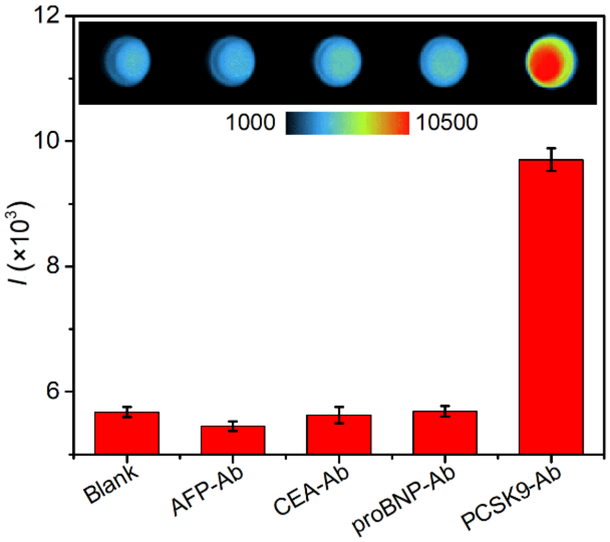

Supplementary Figure 9. Specificity of CLA at 1 μg mL^{-1} PCSK9-Ab, AFP-Ab, CEA-Ab, and proBNP-Ab. Error bars were estimated from three parallel experiments.

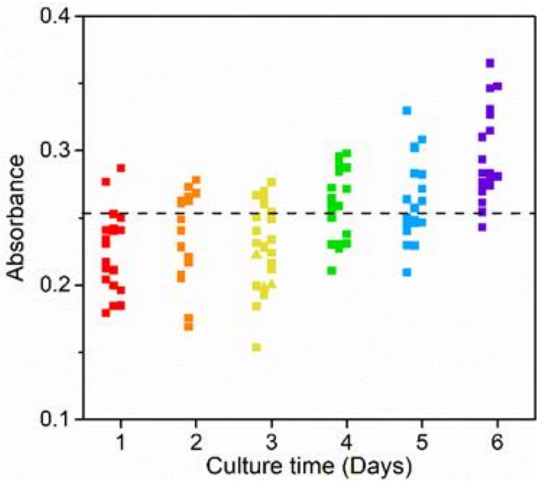

Supplementary Figure 10. ELISA of PCSK9-Ab secreted from 6A6 hybridoma cells with different culture times. Black dash line represents background.

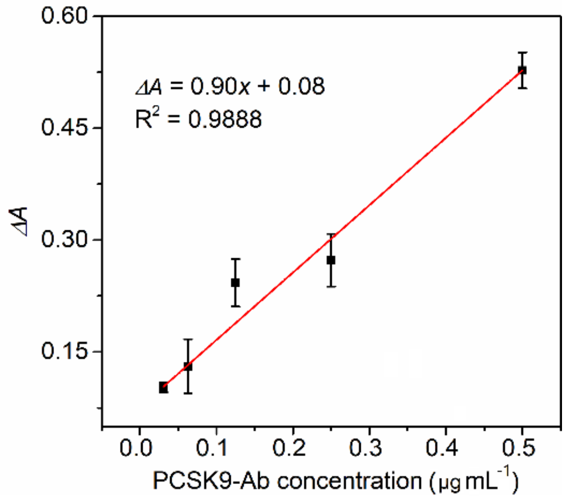

Supplementary Figure 11. ELISA calibration curve for PCSK9-Ab. *ΔA* refers to the signal by subtracting the absorption signal to background. Error bars were estimated from three parallel experiments.

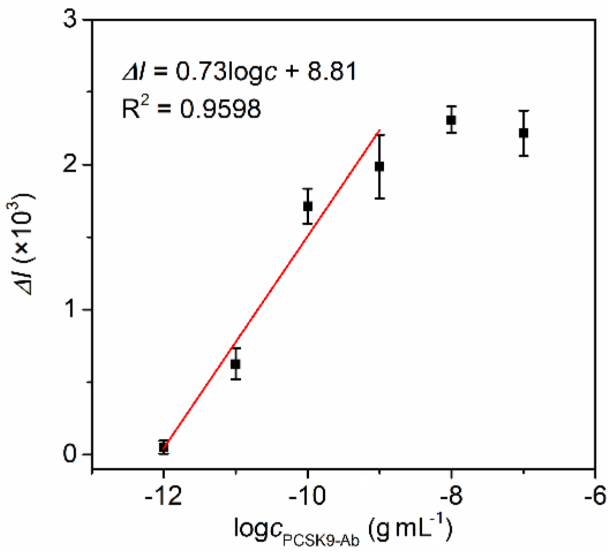

Supplementary Figure 12. CLA calibration curve for PCSK9-Ab in DMEM culture medium. *ΔI* refers the relative chemiluminescent signal after subtracting the background, measured in 10 mM pH 7.2 Tris-HCl (50 μL) containing 0.2 μg mL^{-1} HRP-Cu^{2+}, 0.2 mM luminol and 1 mM H_2O_2 after adding the reaction mixtures (20 μL) for 5 min. The reaction mixtures contain 10 ng mL^{-1} PCSK9-DNA probes, 0.1 μM block-primer, 0.3 μM padlock, 0.625 U μL^{-1} splint R, 0.025 U μL^{-1} phi 29, 1.0 mM BSA, 2.0 mM dNTP and different concentrations of PCSK9-Ab (DMEM diluted) with 1-h incubation at 37 °C. Error bars were estimated from three parallel experiments.

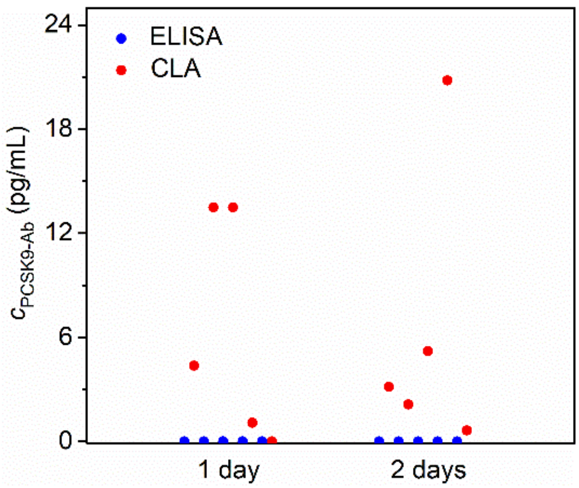

Supplementary Figure 13. Comparison of ELISA and CLA for detection of PCSK9-Ab secreted from 6A6 hybridoma cells with culture for 1 and 2 days.

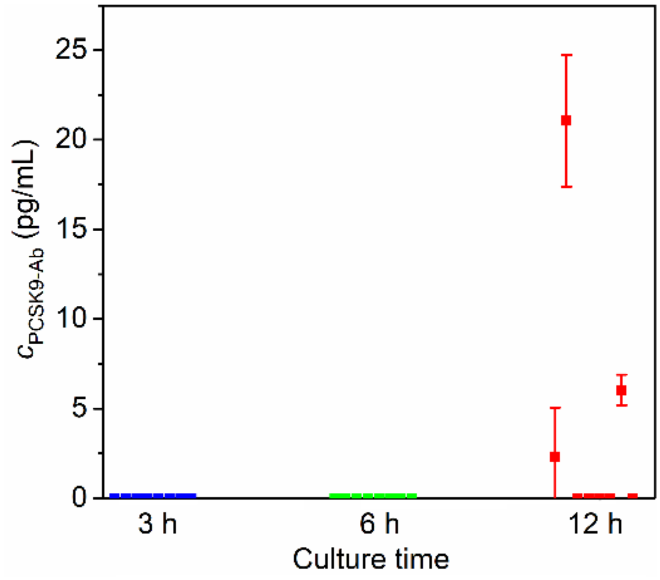

Supplementary Figure 14. CLA of PCSK9-Ab secreted from single hybridoma cell with culture for 3, 6 and 12 hours. Error bars were estimated from three parallel experiments.

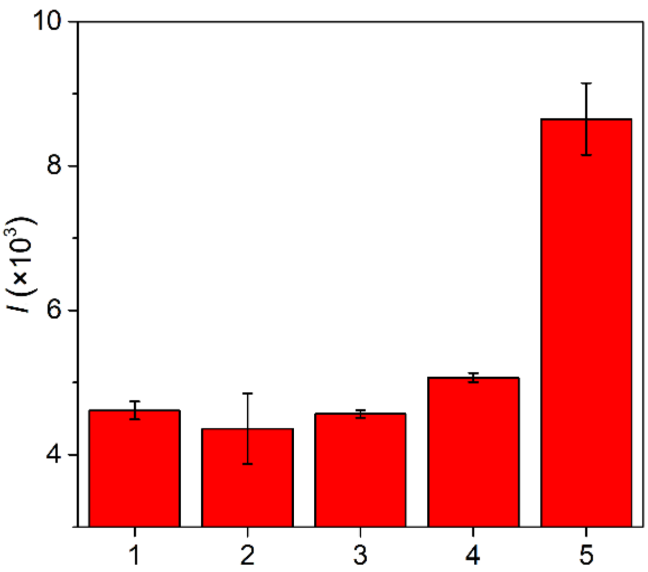

Supplementary Figure 15. CLA of PCSK9-Ab in culture medium of cells secreting or not secreting PCSK9-Ab with culture for 3 days. (1) blank medium without cells, (2-5) culture medium of Hela cells (2), 3H2 hybridoma cells (3), nonsecretor hybridoma cells (4) and 6A6 hybridoma cells (5). Error bars were estimated from three parallel experiments.

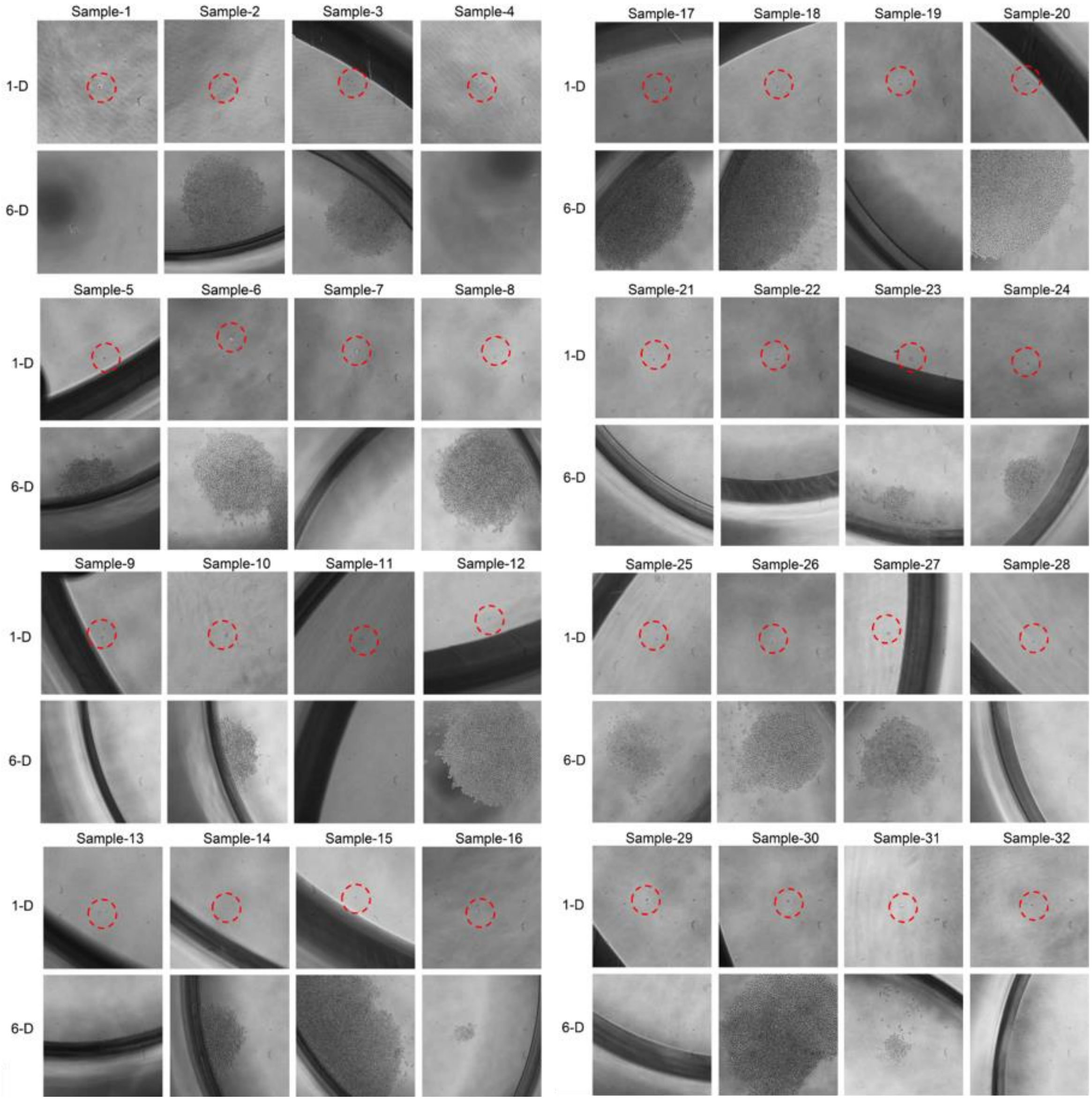

Supplementary Figure 16. Bright-field microscopic images of 32 single hybridoma cells after 1- and 6-day culture.

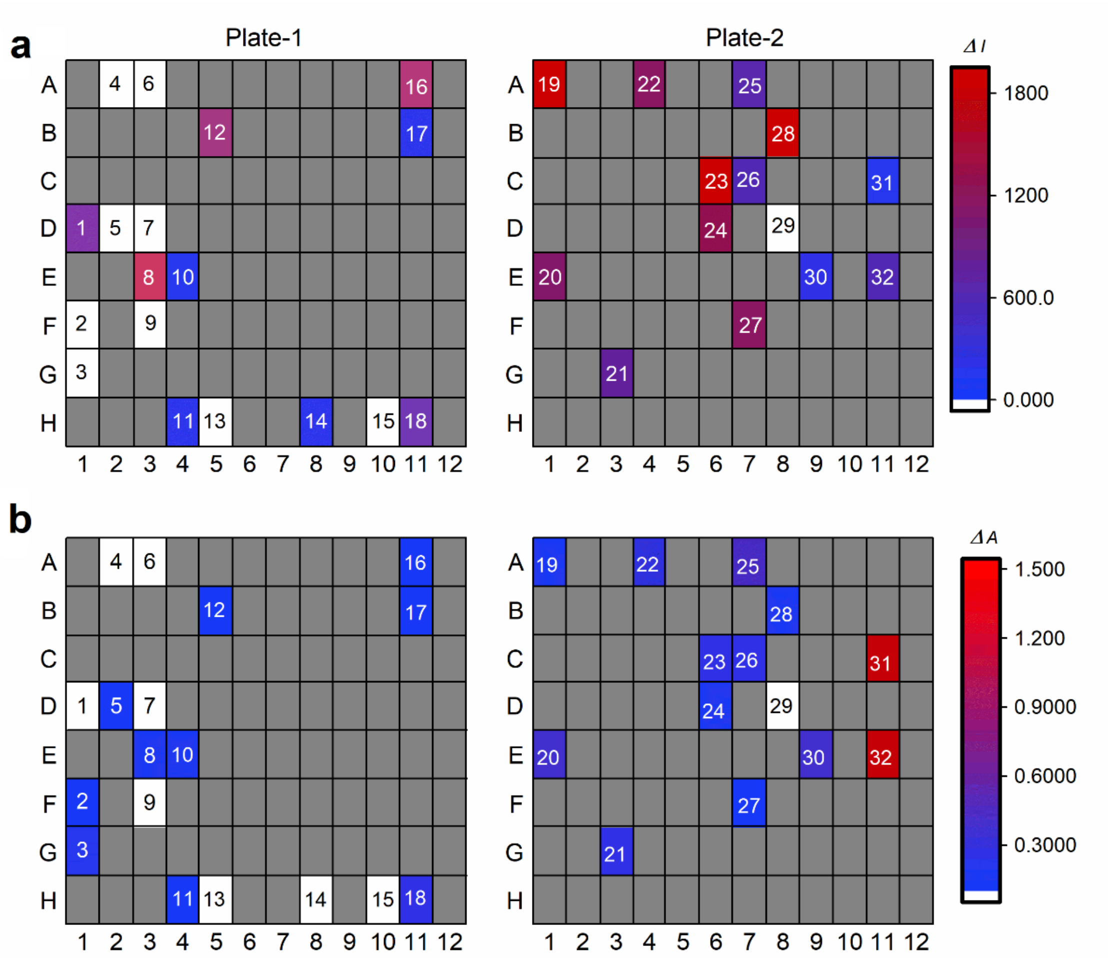

Supplementary Figure 17. Comparison of CLA and ELISA for screening of specific hybridoma cells. **a** *ΔI* and **b** *ΔA* for CLA and ELISA of PCSK9-Ab in supernatants of 32 single hybridoma cell samples distributed on 96-well plates at 1-day (**a**) and 6-day (**b**) culture. Gray squares represent empty wells.

Supplementary References

1. Ranallo, S., Rossetti, M., Plaxco, K. W., Vallee-Belisle, A. & Ricci, F. A modular, DNA-based beacon for single-step fluorescence detection of antibodies and other proteins. *Angew. Chem. Int. Ed*. **54,** 13214–13218 (2015).
2. Rossetti, M. et al. Allosteric DNA nanoswitches for controlled release of a molecular cargo triggered by biological inputs. *Chem. Sci.* **8,** 914–920 (2017).
3. Peng, Y., Li, X., Yuan, R. & Xiang, Y. Steric hindrance inhibition of strand displacement for homogeneous and signal-on fluorescence detection of human serum antibodies. *Chem. Commun.* **52,** 12586–12589 (2016).
4. Porchetta, A. et al. Programmable nucleic acid nanoswitches for the rapid, single-step detection of antibodies in bodily fluids. *J. Am. Chem. Soc.* **140,** 947–953 (2018).
5. Ranallo, S., Sorrentino, D. & Ricci, F. Orthogonal regulation of DNA nanostructure self-assembly and disassembly using antibodies. *Nat. Commun.* **10,** 5509–5518 (2019).
6. Li, N. et al. Proximity-induced hybridization chain assembly with small-molecule linked DNA for single-step amplified detection of antibodies. *Chem. Commun.* **55,** 4387–4390 (2019).
7. Mahshid, S. S., Mahshid, S., Vallee-Belisle, V. & Kelley, S. O. Peptide-mediated electrochemical steric hindrance assay for one step detection of HIV antibodies. *Anal. Chem.* **91,** 4943–4947 (2019).
8. Chen, Z. et al. Proximity recognition and polymerase-powered DNA walker for one-step and amplified electrochemical protein analysis. *Biosens. Bioelectron.* **128,** 104–112 (2019).

9. Rossetti, M. et al. Harnessing effective molarity to design an electrochemical DNA-based platform for clinically relevant antibody detection. *Angew. Chem. Int. Ed.* **59,** 14973–14978 (2020).

10. Yang, S. S., Jiang, M. H., Chai, Y. Q., Yuan, R. & Zhuo, Y. Application of antibody-powered triplex-DNA nanomachine to electrochemiluminescence biosensor for detection of anti-digoxigenin with improved sensitivity versus cycling strand displacement reaction. *ACS Appl. Mater. Interfaces* **10**, 38648–38655 (2018).

11. Adamson, H. et al. Affimer-enzyme-inhibitor switch sensor for rapid wash-free assays of multimeric proteins. *ACS Sens.* **4,** 3014–3022 (2019).
