# Supplementary material for: Chemiluminescent screening of specific hybridoma cells via a proximity-rolling circle activated enzymatic switch
Source: Commun Biol. 2022 Apr 4;5:308. doi: 10.1038/s42003-022-03283-2 (PMC8979942; doi:10.1038/s42003-022-03283-2)
Supplement: Supplementary file 3 — Description of Additional Supplementary Files [file 42003_2022_3283_MOESM3_ESM.pdf]

# Description of Additional Supplementary Files

**File name:** Supplementary Data 1
**Description:** Source data underlying the graphs presented in the main figures.
