# Supplementary material for: Chemiluminescent screening of specific hybridoma cells via a proximity-rolling circle activated enzymatic switch
Source: Commun Biol. 2022 Apr 4;5:308. doi: 10.1038/s42003-022-03283-2 (PMC8979942; doi:10.1038/s42003-022-03283-2)
Supplement: Supplementary file 5 — Reporting Summary [file 42003_2022_3283_MOESM5_ESM.pdf]

nature portfolio

Corresponding author(s): Huangxian Ju

Last updated by author(s): Feb 16, 2022

Reporting Summary

Nature Portfolio wishes to improve the reproducibility of the work that we publish. This form provides structure for consistency and transparency in reporting. For further information on Nature Portfolio policies, see our Editorial Policies and the Editorial Policy Checklist.

Statistics

For all statistical analyses, confirm that the following items are present in the figure legend, table legend, main text, or Methods section.

n/a	Confirmed	
☐	☒	The exact sample size (n) for each experimental group/condition, given as a discrete number and unit of measurement
☐	☒	A statement on whether measurements were taken from distinct samples or whether the same sample was measured repeatedly
☒	☐	The statistical test(s) used AND whether they are one- or two-sided<br>*Only common tests should be described solely by name; describe more complex techniques in the Methods section.*
☒	☐	A description of all covariates tested
☒	☐	A description of any assumptions or corrections, such as tests of normality and adjustment for multiple comparisons
☒	☐	A full description of the statistical parameters including central tendency (e.g. means) or other basic estimates (e.g. regression coefficient) AND variation (e.g. standard deviation) or associated estimates of uncertainty (e.g. confidence intervals)
☒	☐	For null hypothesis testing, the test statistic (e.g. F, t, r) with confidence intervals, effect sizes, degrees of freedom and P value noted<br>*Give P values as exact values whenever suitable.*
☒	☐	For Bayesian analysis, information on the choice of priors and Markov chain Monte Carlo settings
☒	☐	For hierarchical and complex designs, identification of the appropriate level for tests and full reporting of outcomes
☒	☐	Estimates of effect sizes (e.g. Cohen's d, Pearson's r), indicating how they were calculated

Our web collection on statistics for biologists contains articles on many of the points above.

Software and code

Policy information about availability of computer code

Data collection	no software was used.
Data analysis	no software was used.

For manuscripts utilizing custom algorithms or software that are central to the research but not yet described in published literature, software must be made available to editors and reviewers. We strongly encourage code deposition in a community repository (e.g. GitHub). See the Nature Portfolio guidelines for submitting code & software for further information.

Data

Policy information about availability of data

All manuscripts must include a data availability statement. This statement should provide the following information, where applicable:

- Accession codes, unique identifiers, or web links for publicly available datasets
- A description of any restrictions on data availability
- For clinical datasets or third party data, please ensure that the statement adheres to our policy

The datasets generated during and/or analysed during the current study are available from the corresponding author on reasonable request.

Field-specific reporting

Please select the one below that is the best fit for your research. If you are not sure, read the appropriate sections before making your selection.

☒ Life sciences ☐ Behavioural & social sciences ☐ Ecological, evolutionary & environmental sciences

For a reference copy of the document with all sections, see nature.com/documents/nr-reporting-summary-flat.pdf

Life sciences study design

All studies must disclose on these points even when the disclosure is negative.

Sample size	For linear test, error bars were estimated from three parallel experiments. As for assay comparison between ELISA and CLA, 25 samples were randomly collected at different days after cell culture. In CL screening test, 32 single hybridoma cell samples distributed in individual wells were tested by both CLA and ELISA.
Data exclusions	While counting the matching rate of CLA, one sample was excluded considering the apoptosis of cells.
Replication	The recovery test of CLA was executed.
Randomization	Specific hybridoma cells was distributed on 96-well plates by limiting dilution and 25 samples was randomly selected for assay comparison between ELISA and CLA. As for cell screening, after hybridoma cells was distributed on 96-well plates by limiting dilution, all samples were collected for analysis.
Blinding	In the screening of specific hybridoma cells, the investigators were blinded during data collection and analysis.

Reporting for specific materials, systems and methods

We require information from authors about some types of materials, experimental systems and methods used in many studies. Here, indicate whether each material, system or method listed is relevant to your study. If you are not sure if a list item applies to your research, read the appropriate section before selecting a response.

Materials & experimental systems

n/a	Involved in the study	
☐	☒	Antibodies
☐	☒	Eukaryotic cell lines
☒	☐	Palaeontology and archaeology
☒	☐	Animals and other organisms
☒	☐	Human research participants
☒	☐	Clinical data
☒	☐	Dual use research of concern

Methods

n/a	Involved in the study	
☒	☐	ChIP-seq
☒	☐	Flow cytometry
☒	☐	MRI-based neuroimaging

Antibodies

Antibodies used	PCSK9 monoclonal antibody was used in this study, which was researched, developed and supplied by FANTIBODY (Chongqing, China). The clone name of the PCSK9 antibody is 6A6, and lot number is 20190531. The catalog number can not be found because this antibody is not currently commercially available.
Validation	The PCSK9 monoclonal antibody used in this study can bind to human recombinant PCSK9 protein, and its subtype is IgG2b. There is no validation on its application yet.

Eukaryotic cell lines

Policy information about cell lines

Cell line source(s)	SP2/0 myeloma cells and Hela cells was used in this study. PCSK9 hybridoma cells was obtained by the fusion of SP2/0 myeloma cells with spleen cells from immunized BALB/c female mice, and Hela cells was purchase from KeyGEN BioTECH Corp., Ltd (Jiangsu, China).
Authentication	none of the cell lines used were authenticated.
Mycoplasma contamination	the cell lines were not tested for mycoplasma contamination.

Commonly misidentified lines<br>(See ICLAC register)	no other cell lines used in this study.
